# Clinical Indicators of Hepatotoxicity in Newly Diagnosed Acute Promyelocytic Leukemia Patients Undergoing Arsenic Trioxide Treatment

**DOI:** 10.1007/s12011-023-03676-2

**Published:** 2023-04-25

**Authors:** Zhuo Zhang, Shunji Zhang, Fan Zhang, Qian Zhang, Hong Wei, Ruolin Xiu, Yanhong Zhao, Meijuan Sui

**Affiliations:** 1 Central Laboratory, the First Affiliated Hospital of Harbin Medical University, No. 23 Youzheng Street, Harbin, 150001 Heilongjiang China; 2 Department of Hematology, Southern University of Science and Technology Hospital, Shenzhen, China; 3 Department of Hematology, the First Affiliated Hospital of Harbin Medical University, Harbin, China; 4 Key Laboratory of Hepatosplenic Surgery, Ministry of Education, The First Affiliated Hospital of Harbin Medical University, Harbin, China

**Keywords:** Arsenic trioxide, Hepatotoxicity, Acute promyelocytic leukemia, Indicators

## Abstract

Arsenic trioxide (ATO)-induced hepatotoxicity is often observed in acute promyelocytic leukemia (APL) patients and decreases therapeutic effect of ATO. Thus, concerns over hepatotoxicity have been raised. The aim of this study was to explore some noninvasive clinical indicators that can be used to guide the individualized application of ATO in the future. APL patients treated with ATO were identified retrospectively via electronic health records at our hospital from August 2014 through August 2019. APL patients without hepatotoxicity were selected as controls. The association between putative risk factors and ATO-induced hepatotoxicity was estimated with ORs and 95% CIs, which were calculated using the chi-square test. The subsequent multivariate analysis was performed using logistic regression analysis. In total, 58.04% of patients experienced ATO-induced hepatotoxicity during the first week. Elevated hemoglobin (OR 8.653, 95% CI, 1.339–55.921), administration of nonprophylactic hepatoprotective agents (OR 36.455, 95% CI, 7.409–179.364), non-single-agent ATO to combat leukocytosis (OR 20.108, 95% CI, 1.357–297.893) and decreased fibrinogen (OR 3.496, 95% CI, 1.127–10.846) were found to be statistically significant risk factors for ATO-induced hepatotoxicity. The area under the ROC curve values were 0.846 for “overall ATO-induced hepatotoxicity” and 0.819 for “early ATO-induced hepatotoxicity.” The results revealed that hemoglobin ≥ 80 g/L, nonprophylactic hepatoprotective agents, and non-single-agent ATO and fibrinogen < 1 g/L are risk factors for ATO-induced hepatotoxicity in newly diagnosed APL patients. These findings can enhance the clinical diagnosis of hepatotoxicity. Prospective studies should be performed in the future to validate these findings.

## Introduction

Arsenic trioxide (As_2_O_3_, ATO) was first introduced as a treatment for acute promyelocytic leukemia (APL) patients at our hospital in the 1970s. Since then, it has been established as an effective therapeutic agent for acute promyelocytic leukemia patients, yielding a complete remission (CR) rate of greater than 90%, even in relapsed patients [1, 2]. However, ATO has some toxic effects and is associated with serious side effects in a subset of patients [3–5].

ATO-induced hepatotoxicity is often observed and reduces the therapeutic effect of ATO, and substantial concerns have been raised over hepatotoxicity in APL patients undergoing ATO treatment [6]. It is therefore necessary to identify the influencing factors and severity of hepatotoxicity. Few studies have examined the indicators of ATO-induced hepatotoxicity, and even basic research on risk factors is lacking. In this retrospective study, the incidence and characteristics of ATO-induced hepatotoxicity were analyzed in newly diagnosed APL patients initially treated with ATO. Subgroup analyses of patients with ATO-induced hepatotoxicity were carried out and included an overall ATO-induced hepatotoxicity patient group and an early ATO-induced hepatotoxicity patient group. This study provides evidence-based guidance for the early prediction of the timing and intensity of ATO-induced hepatotoxicity by combining basic clinical indicators and laboratory parameters, thereby maximizing the effectiveness of ATO through targeted intervention.

To date, our study has the largest sample size of newly diagnosed APL patients who were initially treated with ATO. The specific aim of the work was to explore some noninvasive clinical indicators, contribute to the comprehensive analysis of hepatotoxicity and provide guidance for be used to guide the individualized application of ATO in the future. Prospective studies should be performed in the future to validate these findings.

## Materials and Methods

### Patient Selection

**Fig. 1:**
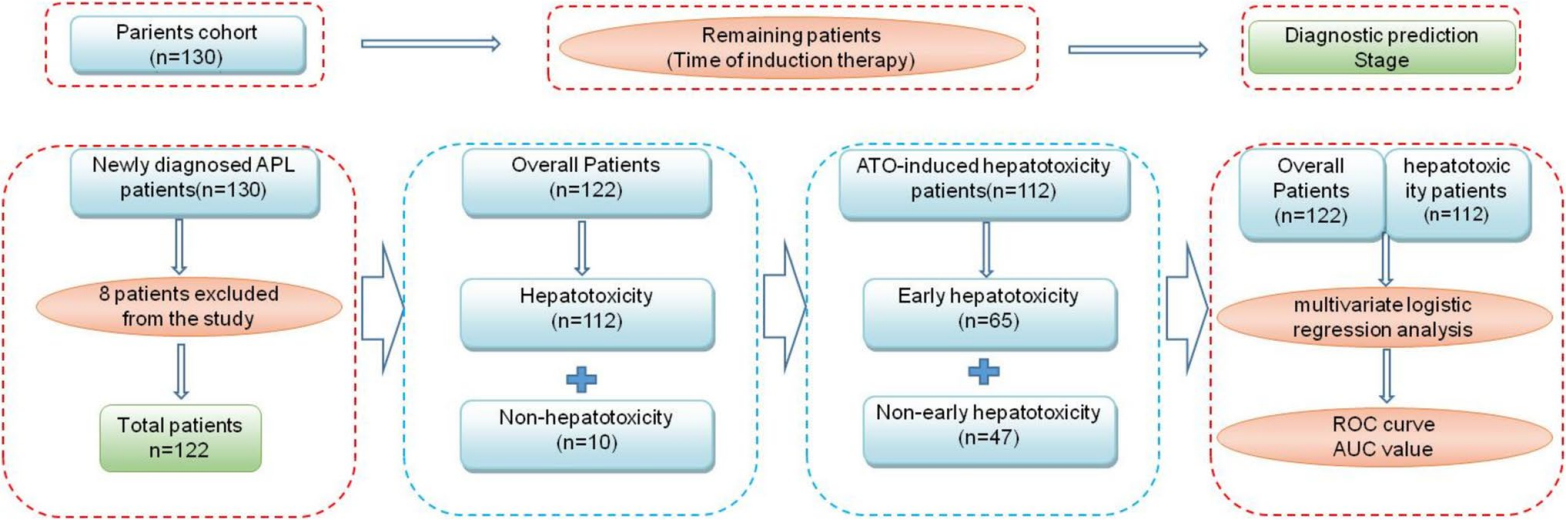
Overviews of the research schedule. ATO, arsenic trioxide; APL, acute promyelocytic leukemia. During induction therapy, enrolled patients received ATO for 4 weeks. Chi-square tests and multivariate logistic regression analysis were used to examine ATO-induced hepatotoxicity. Eight patients were excluded from the study: 4 patients died early (the first week); and the other 4 patients were excluded because their personal economic situation precluded them from receiving ATO treatment, and thus, they could not be evaluated for the hepatotoxicity of As_2_O_3_

The study protocol was approved by the Medical Ethics Committee of the First Affiliated Hospital of Harbin Medical University (file has been attachment uploaded). The Ethics Committee has confirmed that this study was exempt from the need for informed consent of the patients due to the retrospective, observational nature of the study. P and patient data were identity remained anonymous. Thus, the study did not include confidential data and interventions. The participants in this retrospective study were 130 consecutive newly diagnosed APL patients undergoing ATO induction treatment from August 2014 to August 2019 in our hospital. None of the patients relapsed and they were initially treated at the First Affiliated Hospital of Harbin Medical University. For all the patients, the inclusion criteria were as follows: ① the diagnosis was confirmed by the presence of t (15; 17) and/or the PML/RARα fusion gene; ② the APL patients were newly diagnosed and first treated; and ③ ATO was used as first-line induction therapy. The exclusion criteria were as follows: ① a previous history of hepatitis, excessive drinking and other diseases affecting hepatobiliary function; ② abnormal renal function, liver function or electrocardiographic findings; ③ a history of arsenic exposure; and ④ unwillingness to join the study. The medical records of the 130 patients were extracted and recorded from the electronic medical records. All patients were treated in the hematology department of our hospital (First Affiliated Hospital of Harbin Medical University). The patients were treated by hematology physicians in our hospital. No ethical approval is required (Fig. 1).

### Data Collection

Clinical and laboratory data were collected from medical records after approval by the local institutional Helsinki ethics committee. The data included age, sex, WBC count, platelet count, hemoglobin level, prophylactic hepatoprotective agents, and anti-leukocytosis agents.

### ATO Treatment Protocol

All patients were treated with a continuous slow intravenous infusion of ATO. The ATO solution (10 mg/10 mL) was supplied by Harbin Yida Pharmaceutical Company, dissolved in 500 mL 5% dextrose and administered daily at a dose of 0. 20 mg/kg for children > 6 years old and 0.16 mg/kg for children ≤ 6 years old, with a maximum daily dose of 10 mg. The total ATO dose was infused intravenously over the course > 18 h [7]. Low-dose chemotherapy agents (no more than a standard dose) (adjusted-dose daunorubicin, Ara-c (cytosine arabinoside) or hydroxyurea) were administered to patients with higher leukocyte counts at the time of initial treatment or following administration of As_2_O_3_ to prevent differentiation syndrome (DS). However, the dose of ATO was not decreased throughout the whole process for the subset of patients with leukocytosis.

### Definition and Potential Clinical Indicators of ATO-Induced Hepatotoxicity

In this study, “ATO-induced hepatotoxicity” was defined based on laboratory test results indicating higher than normal levels of the enzymes alanine aminotransferase (ALT), aspartate aminotransferase (AST), or gamma-glutamyltransferase (GGT) in blood samples from patients undergoing ATO as initial treatment for newly diagnosed APL. “Early hepatotoxicity” was defined as ATO-induced hepatotoxicity that occurred within the first week after ATO treatment. Hepatotoxicity was graded using the WHO toxicity grading scale for determining the severity of adverse events. Liver function was monitored weekly. For patients with impaired liver function, comprehensive monitoring was provided, the necessary supportive therapy was administered until the end of the treatment and the patients achieved CR. The “prophylactic application of hepatoprotective agents” is defined as the application of a hepatoprotective agent prior to the time point of hepatotoxicity occurrence.

Based on clinical experience and previous relevant literature, eight basic indicators or laboratory parameters were selected for further analysis, all of which were noninvasive and easy to observe: age, sex, white blood cell count (WBC), platelet count (Plt), hemoglobin (Hb), and fibrinogen (FIB), whether the patients received hepatoprotective drugs and whether small doses of low-dose agents were effective against leukocytosis. The hepatotoxic patients and nonhepatotoxic patients were compared across all patients, while early hepatotoxic patients and nonearly hepatotoxic patients were compared across hepatotoxic patients. These factors have been previously investigated for their predictive value for ATO-induced hepatotoxicity in different subgroups. The factors related to overall hepatotoxicity and those related to early hepatotoxicity were further determined.

### Statistical Analysis

All data were analyzed using SPSS 17.0 software and GraphPad Prism 6. All tests were two-sided, and a *P*-value of less than 0.05 was considered to indicate statistical significance. To make a simpler and more convenient assessment in clinical practice, all continuous variables were dichotomized into binary variables. The cutoff points for the indicators were set according to clinical experience and the corresponding literature. Chi-square or Fisher’s exact test (*n* < 5) was applied for univariate analysis. The subsequent multivariate analysis was performed using logistic regression analysis. Receiver operating characteristic (ROC) curve analysis and the area under the ROC curve (AUC) were used to evaluate the ability of the prediction models to screen for overall hepatotoxicity or early hepatotoxicity.

## Results

### Clinical Characteristics of the Study Populations

Patients met the exclusion criteria (patients had baseline hepatitis/excessive drinking/hepatobiliary abnormality, etc.) were not included. Eight patients were excluded from the 130-patient study because they could not continue treatments during the early stage: 4 patients died early (the first week), but none of these patients developed liver dysfunction before their death; and the other 4 patients were excluded from further analyses in this study; because their personal economic situation precluded them from receiving ATO treatment, and thus, they could not be evaluated for the hepatotoxicity of As_2_O_3_. In total, 122 patients with newly diagnosed APL undergoing ATO treatment were included in this retrospective study. Among them, there were 112 patients with hepatotoxicity, and these patients with hepatotoxicity did not receive any complementary medicine products and/or drugs that might induce hepatotoxicity except ATO. In addition, the basic characteristics of 112 patients with early ATO-induced hepatotoxicity were analyzed. Information was extracted from the electronic medical records by individual chart review. The main clinical characteristics of the patients are listed in Table 1.

**Table 1:** Demographic and baseline clinical characteristics of the patients in the study

Clinical characteristics	Overall patients Median (range) or no. (%)	Hepatotoxic patients Median (range) or no. (%)
Total	122	112
Age, years	40 (7–81)	40 (7–81)
> 50	39 (31.97%)	36 (32.14%)
≤ 50	83 (68.03%)	76 (67.86%)
Sex	_	_
Female	57 (46.72%)	53 (47.32%)
Male	65 (53.28%)	59 (52.68%)
WBC count, × 10^9^/L	2.755 (0.23–136.9)	2.545 (0.34–136.9)
≤ 10	97 (79.51%)	90 (80.36%)
> 10	25 (20.49%)	22 (19.64%)
Platelet count, × 10^9^/L	24.355 (3.32–222.8)	23.69 (3.32–222.8)
≥ 30	49 (40.16%)	44 (39.29%)
< 30	73 (59.84%)	68 (60.71%)
Hemoglobin level, g/L	79.985 (40–154.3)	80.015 (40–154.3)
≥ 80	61 (50.00%)	58 (51.79%)
< 80	61 (50.00%)	54 (48.21%)
Fibrinogen level, g/L	1.215 (0.3–5.45)	1.255 (0.3–5.45)
≥ 1	80 (65.57%)	75 (66.96%)
< 1	42 (34.43%)	37 (33.04%)
Prophylactic hepatoprotective agents	_	_
Yes	28 (22.95%)	21 (18.75%)
No	94 (77.05%)	91 (81.25%)
Anti-leukocytosis agents	_	_
Single ATO^*^	76 (62.30%)	68 (60.71%)
ATO + chemotherapy	46 (37.70%)	44 (39.29%)

*ATO, arsenic trioxide

Among all patients with ATO-induced hepatotoxicity and those with early ATO-induced hepatotoxicity, the majority of patients (accounting for a higher percentage) were young and male; they did not have elevated leukocyte, fibrinogen, or hemoglobin levels or a decreased platelet count; and they received nonprophylactic application of hepatoprotective drugs (or not) combined with low-dose chemotherapy agents against leukocytosis (single dose of ATO). The anti-leukocytosis agents used were mainly low-dose anthracyclines and hydroxyurea (both are administered at a lower dose than the standard chemotherapy dose and for a shorter duration).

### Occurrence and Intensity of ATO-Induced Hepatotoxicity

**Fig. 2:**
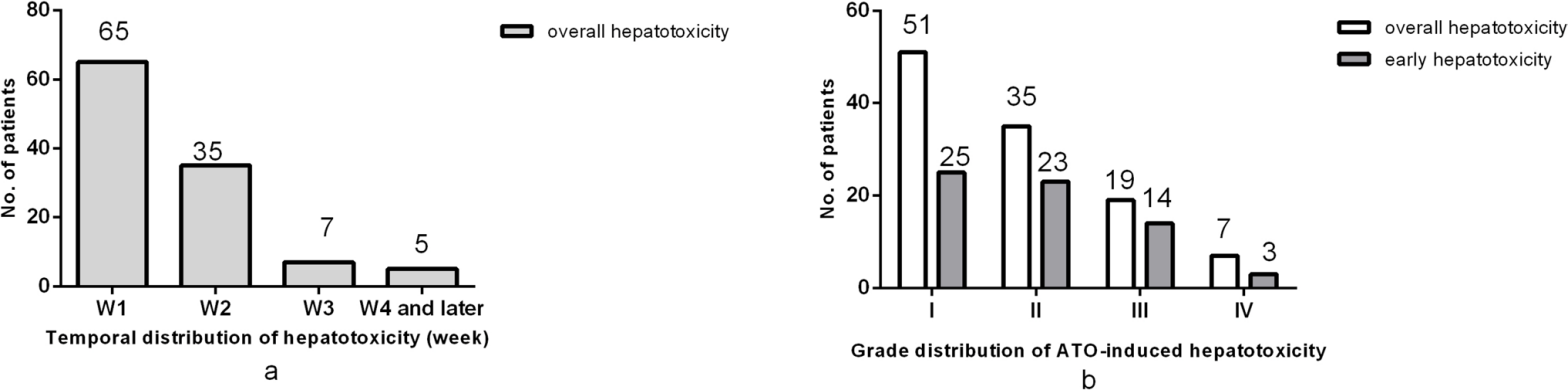
The temporal and severity distribution of ATO-induced hepatotoxicity in APL patients. **a** The temporal and severity distribution of ATO-induced hepatotoxicity in all APL patients. *W1,* the 1st week; *W2,* the 2nd week; *W3*, the 3rd week; ≥ *W4*, during or after the 4th week of ATO treatment. **b** The severity distribution of ATO-induced hepatotoxicity in ATO-induced hepatotoxicity.* I*, grade 1 toxicity; *II*, grade 2 toxicity; *III*, grade 3 toxicity; *IV*, grade 4 toxicity according to the WHO toxicity grading scale; ATO, arsenic trioxide; APL, acute promyelocytic leukemia

The study cohort consisted of 122 enrolled patients, and 112 (91.8%) patients had laboratory evidence of hepatotoxicity during ATO induction therapy. The median age was 40 years (range 7–81 years). ATO-induced hepatotoxicity appeared at different time points and to different degrees. In addition, the time points of ATO-induced hepatotoxicity differed. Sixty-five cases occurred during the first week of ATO treatment, accounting for 58.04% of all patients with ATO-induced hepatotoxicity; 47 cases occurred during the following induction treatment period (later than the 1st week), including 35 cases in the 2nd week, 7 cases in the 3rd week, and 5 cases in the ≥ 4th week. The results showed that most of the hepatotoxicity cases occurred during the first 1 to 2 weeks of ATO treatment, with 89.29% of the patients affected (Fig. 2a). The number of hepatotoxic patients gradually decreased over the treatment time, and the median time to hepatotoxicity appeared on the 6th day (range 1–43 days).

On the other hand, according to the WHO toxicity grading scale for determining the severity of adverse events, the severity of ATO-induced hepatotoxicity distribution was different in our study patients: 51 cases were grade I toxicity, 35 cases were grade II toxicity, 19 cases were grade III toxicity, and 7 cases were grade IV toxicity. A total of 76.79% of the liver toxicity cases were grades I and II (Fig. 2b). The number of affected patients gradually decreased as the severity of hepatotoxicity increased. Hepatotoxicity is generally transient, the duration generally does not exceed 2 weeks (100/112, 89.29%), and all patients showed no obvious clinical symptoms of hepatotoxicity.

### Univariate Analysis of the Clinical Parameters for ATO-Induced Hepatotoxicity

Basic clinical indicators or laboratory parameters were included in the univariate analysis of indicators of ATO-induced hepatotoxicity. The chi-square test was performed to compare hepatotoxic patients and nonhepatotoxic patients (Table 2).

**Table 2:** Univariate analysis of predictive factors for ATO-induced hepatotoxicity

Clinical characteristics	All Patients (*N* = 122)		
	Cases	Controls	
	Hepatotoxicity, no. (%)	Nonhepatotoxicity, no. (%)	*P*
Total	112 (91.80%)	10 (8.20%)	—
Age, years			
> 50	36 (92.31%)	3 (7.69%)	0.889^a^
≤ 50	76 (91.57%)	7 (8.43%)	
Sex			
Female	53 (92.98%)	4 (7.02%)	0.749^a^
Male	59 (90.77%)	6 (9.23%)	
WBC count, × 10^9^/L			
≤ 10	90 (92.78%)	7 (7.22%)	0.426^a^
> 10	22 (88%)	3 (12%)	
Platelet count, × 10^9^/L			
≥ 30	44 (89.80%)	5 (10.20%)	0.508
< 30	68 (93.15%)	5 (6.85%)	
Hemoglobin level, g/L			
≥ 80	58(95.08%)	3 (4.92%)	0.323^a^
< 80	54 (88.52%)	7 (11.48%)	
Fibrinogen level, g/L			
≥ 1	75 (93.75%)	5 (6.25%)	0.279
< 1	37 (88.10%)	5 (11.90%)	
Prophylactic hepatoprotective agents			
Yes	22 (78.57%)	6 (21.43%)	0.01**^a^
No	90 (95.74%)	4 (4.26%)	
Anti-leukocytosis agents			
Single ATO	68 (89.47%)	8 (10.53%)	0.317^a^
ATO + chemotherapy	44 (95.65%)	2 (4.35%)	

Bold values and ^*^ are statistically significant (*P* < 0.05); Bold values and ^*^ are statistically significant (*P* < 0.01); *ATO*, arsenic trioxide; a, *P*-values for categorical variables are from Fisher’s exact test (*n* < 5)

The univariate analysis results showed that “no prophylactic hepatoprotective agents” was a risk factor for ATO-induced hepatotoxicity (*P* = 0.01). The other clinical parameters (age, sex, WBC count, platelet count, hemoglobin level, and agents against leukocytosis) were not statistically significant indicators of the occurrence of ATO-induced hepatotoxicity in the univariate analysis.

### Univariate Analysis of the Time of Occurrence of ATO-Induced Hepatotoxicity

Next, univariate analysis for the time of occurrence of ATO-induced hepatotoxicity was performed. Because the risk factors and treatment principles might differ for hepatotoxicity within 7 days and within 8–30 days, we also assessed risk factors for hepatotoxicity during these two time periods in this study. The results of univariate analysis for the time of occurrence of ATO-induced hepatotoxicity are shown in Table 3.

**Table 3:** Univariate analysis of the time of occurrence of ATO-induced hepatotoxicity

Clinical characteristics	Hepatotoxic patients (*N* = 112)		
	Early hepatotoxic patients (within 7 days), no. (%)	Nonearly hepatotoxic patients (within 8–28 days), no. (%)	*P*
Total	65 (58.04%)	47 (41.96%)	
Age, years			
> 50	18 (50%)	18 (50%)	0.236
≤ 50	47 (61.84%)	29 (38.16%)	
Sex			
Female	28 (52.83%)	25 (47.17%)	0.290
Male	37 (62.71%)	22 (37.29%)	
WBC count, × 10^9^/L			
≤ 10	48 (53.33%)	42 (46.67%)	0.041*
> 10	17 (77.27%)	5 (22.73%)	
Platelet count, × 10^9^/L			
≥ 30	23 (52.27%)	21 (47.73%)	0.320
< 30	42 (61.76%)	26 (38.24%)	
Hemoglobin level, g/L			
≥ 80	37 (63.79%)	21 (36.21%)	0.201
< 80	28 (51.85%)	26 (48.15%)	
Fibrinogen level, g/L			
≥ 1	37 (49.33%)	38 (50.67%)	0.008**
< 1	28 (75.68%)	9 (24.32%)	
Prophylactic hepatoprotective agents			
Yes	4 (18.18%)	18 (81.82%)	0.000**
No	61 (67.78%)	29 (32.22%)	
Anti-leukocytosis agents			
Single ATO	34 (50.00%)	34 (50.00%)	0.032*
ATO + chemotherapy	31 (70.45%)	13 (29.55%)	

Bold values and ^*^ are statistically significant (*P* < 0.05); Bold values and ^*^ are statistically significant (*P* < 0.01); *ATO*, arsenic trioxide; a, *P*-values for categorical variables are from Fisher’s exact test (*n* < 5)

The chi-square test was performed between early hepatotoxic patients and non-early hepatotoxic patients in hepatotoxic patients. The prognostic risk factors for “overall ATO-induced hepatotoxicity” and “early ATO-induced hepatotoxicity” were the same and included no prophylactic hepatoprotective agents in univariate analysis (*P* = 0.004 and *P* = 0.000, respectively) (Tables 2 and 3).The reason may be related to the large proportion of patients with early hepatotoxicity among all hepatotoxic patients. Notably, some risk factors were not statistically significant in the overall hepatotoxicity analysis but were in the early hepatotoxicity analysis (Table 3). “Early ATO-induced hepatotoxicity” was associated with WBC count (*P* = 0.041), fibrinogen level (*P* = 0.008), absence of prophylactic hepatoprotective agents (*P* = 0.000) and combination with low-dose chemotherapy agents against leukocytosis (*P* = 0.032).

### Multivariate Analysis of Indicators for ATO-Induced Hepatotoxicity

In the multivariate analysis, the same independent prognostic risk factors were no prophylactic hepatoprotective agents combined with low-dose chemotherapy agents against leukocytosis for both overall hepatotoxic patients and early hepatotoxic patients (Table 4). Notably, the study revealed statistically significant inconsistencies across some risk factors in both overall hepatotoxic patients and early hepatotoxic patients. The prognostic risk factors for overall hepatotoxicity included no prophylactic hepatoprotective agents (*P* = 0.004), combined with low-dose chemotherapy agents against leukocytosis (*P* = 0.029) and higher HB levels (*P* = 0.023), while early hepatotoxicity was associated with lower fibrinogen levels (*P* = 0.030), no prophylactic hepatoprotective agents (*P* = 0.000) and combined with low-dose chemotherapy agents against leukocytosis (*P* = 0.013) (Table 4). Among these prognostic risk factors, odds ratio (OR) values were high, which presented good predictive abilities for both types of hepatotoxicity. Age, sex, and platelet count were not indicators in either subgroup of patients.

**Table 4:** Multivariate analysis of risk factors for hepatotoxicity in APL patients undergoing ATO treatment

Variables	Unfavorable category	Overall patients			Unfavorable category	Early hepatotoxicity		
		OR	95% CI	*P*		OR	95% CI	*P*
Age	≥ 50	2.077	0.377–11.429	0.401	≤ 50	2.058	0.744–5.693	0.164
Fibrinogen level, g/L	≥ 1	3.665	0.685–19.598	0.129	< 1	3.496	1.127–10.846	0.030^*^
Hemoglobin level, g/L	≥ 80	8.653	1.339–55.921	0.023^*^	≥ 80	1.756	0.662–4.659	0.258
Sex	female	2.472	0.434–14.065	0.308	male	2.202	0.820–5.841	0.113
Platelet count, × 10^9^/L	< 30	4.356	0.786–24.141	0.092	< 30	2.373	0.886–6.354	0.086
Prophylactic hepatoprotective agents	No	14.531	2.349–89.879	0.004^**^	No	36.455	7.409–179.364	0.000^**^
Single ATO	No	20.108	1.357–297.893	0.029^*^	No	4.288	1.363–13.492	0.013^*^

*OR*, odds ratio; *CI*, confidence interval; Bold values and ^*^ are statistically significant (*P* < 0.05); bold values and ^**^ are statistically significant (*P* < 0.01); *ATO*, arsenic trioxide

The causes of the difference in indicators between the overall and early hepatotoxicity analyses may be related to the inconsistent distribution of patients in different indicators. For the WBC count, the occurrence of early hepatotoxicity was higher in the WBC > 10 × 10^9^/L group (77.27%) than in the WBC ≤ 10 × 10^9^/L group but was different in overall hepatotoxicity (Tables 2 and 3). Similarly, for fibrinogen level, the incidence of early hepatotoxicity was higher in patients with FIB < 1 g/L (75.68%) than in patients with FIB ≥ 1 g/L. Age, sex, and platelet count were not indicators in either subgroup.

**Fig. 3:**
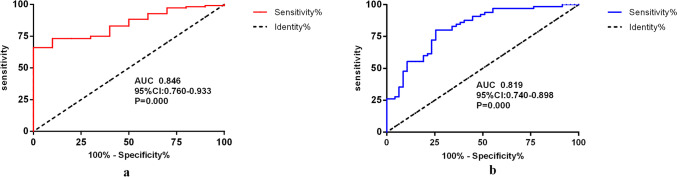
Receiver operating characteristic (ROC) curve for combined variables for hepatotoxicity. **a** ROC curve for combined variables for overall ATO-induced hepatotoxicity, **b** ROC curve for combined variables for early ATO-induced hepatotoxicity; AUC, area under the ROC curve

We generated a receiver operating characteristic (ROC) curve using the dichotomized variables. The area under the ROC curve (AUC) can quantify the predictive ability of the combined variable for overall hepatotoxicity or early hepatotoxicity. The area under the ROC curve (AUC) of the combined variable for overall hepatotoxicity was 0.846 (95% CI, 0.760–0.933; *P* = 0.000) (Fig. 3a), and the ROC curve of the combined risk factors for early hepatotoxicity had an AUC of 0.819(95% CI, 0.740–0.898; *P* = 0.000) (Fig. 3b). The Hosmer–Lemeshow test indicated that the model fit well for both the overall hepatotoxicity and early hepatotoxicity cohorts (χ^2^ = 7.871, df = 8, *P* = 0.446 and χ^2^ = 8.429, df = 8, *P* = 0.393, respectively). All these results indicate that the combined risk factors had considerable predictive value.

## Discussion

Arsenic trioxide is an attractive drug for the treatment of acute promyelocytic leukemia, but increased liver enzymes are common. Previous studies have indicated that prophylactic application of hepatoprotective agents and the temporary discontinuation of ATO are generally administered to patients to reverse elevated liver enzymes. However, these interventions will cause the effective arsenic concentration to be lower than the apoptotic concentration, reducing the curative effect of ATO [8–10]. Reducing ATO-induced hepatotoxicity without decreasing the efficacy is a problem that needs to be urgently solved. It has been reported that regimens with ATO have a higher incidence of hepatotoxicity than regimens without ATO when used for the treatment of APL [4]. Therefore, our research focuses on the incidence and indicators of hepatotoxicity in newly diagnosed APL patients initially treated with ATO. We investigated indicators for the time of occurrence and intensity of ATO-induced hepatotoxicity in the overall and early ATO-induced hepatotoxicity patient groups. This study provides evidence to aid in early predictions of the timing and intensity of ATO-induced hepatotoxicity by combining basic clinical indicators and laboratory parameters so that the effectiveness of ATO can be maximized through targeted intervention. Our study did not examine the influence of underlying diseases of the liver and gallbladder. The metabolism of arsenic in the body is affected by many factors. In general, seafood consumption is prohibited during therapy when arsenic is used for APL patient treatment in our hospital, and interference was also eliminated objectively.

Hepatotoxicity data from APL patients treated with ATO are generally lacking, and the incidences of hepatotoxicity often differ among patients who receive different therapies. Recent literatures were consulted, there are some impressive report in animal model on As_2_O_3_. In the rat model, mitochondria are the first target of arsenic-induced hepatotoxicity, and the mechanism is that antioxidant defense or complex II is involved in mitochondrial dysfunction [11]. Other study has shown that Chk1-p53 pathway is involved in arsenic-induced hepatotoxicity [12]. ATO-induced hepatotoxicity was association with increased antioxidant enzyme, decreased malondialdehyde and ATO-induced hepatocyte apoptosis and inflammatory reaction, increased BCL-2 protein expression and decreased levels of BAX, caspase-3, interleukin-1 β, IL-6, and tumor necrosis factor α [13]. Our study provides novel data on the ATO-induced hepatotoxicity occurrence rate and risk factors in APL patients undergoing ATO treatment. To date, our study includes the largest sample of newly diagnosed and initially treated APL patients with ATO. In the literature, the incidence of hepatotoxicity varies. A study from China showed that the incidence of liver injury in 70 patients with AML was 73.85%, while the incidence of M3 was 65% [14]. This study included patients with multiple treatment options. A 76-patient study in India showed that hepatotoxicity was observed in 65.5% of APL patients treated with single-agent ATO [6]. It has been reported in the literature that the rate of liver dysfunction in APL treated with ATRA alone is approximately 12–30% [15]. The hepatotoxicity incidence of As_2_O_3_ for the treatment of APL was 63.6% [16], and that of ATRA combined with ATO was 65.6% [6]. In our cohort of patients treated with ATO, we found that hepatotoxicity continued to be a common toxicity impacting therapy. During the induction therapy course, more than 90% of patients developed ATO-induced hepatotoxicity, which was significantly higher than that reported in previous studies. This is particularly concerning during the early stage, as early hepatotoxicity was observed in more than half of all cases (58.04%). However, no treatment-related mortality from hepatotoxicity was observed during induction therapy, which is reassuring. At this stage, such a high incidence may be associated with the unique role of ATO; “continuous slow intravenous infusion of ATO” keeps the effective concentration high, which promotes apoptosis and does not induce differentiation [7]. The liver is temporarily unable to metabolize arsenic metabolites within a short time, and a complementary increase in liver enzymes appears.

Liver toxicity was associated with elevated liver enzymes in our study, which is consistent with previous studies. Hepatotoxicity was mostly weak, with majority of cases being grade I/II, which is better than that observed after conventional chemotherapy. Hepatoprotective agents were applied for patients with ATO-induced hepatotoxicity, which included glutathione, ammonium glycyrrhizinate S, diisopropylamine dichloroacetate, magnesium isoglycyrrhizinate, polyene phosphatidylcholine, and bicyclol. The elevated liver enzymes returned to normal levels or decreased significantly in majority of patients with ATO-induced hepatotoxicity at the end of induction therapy. However, with our protocol, majority of ATO-induced hepatotoxicity was mild, and the dose of ATO was not suspended or reduced. Hepatotoxicity improved at the end of induction therapy, which was different from other studies. In our study, all 130 patients were newly diagnosed with APL and underwent ATO induction therapy, and we excluded relapsed or refractory patients. These enrollment criteria can ensure that the study population better reflects the characteristics of ATO-induced hepatotoxicity in induction therapy, but they also lead to a reduction in sample size.

It has been reported that the adverse reactions of ATO may be related to its unique metabolic pattern and direct or indirect effects on different organs [17]. The mechanism of drug-induced liver toxicity has been studied in the past and involves oxidative stress, lipid peroxidation, and calcium overload [18–20]. Although there have been some studies on arsenic toxicity in vivo and in vitro, the mechanisms are not completely understood. Previous studies have focused on the protection from arsenic toxicity, which may be related to the special feature of arsenic-specific toxicity mechanism. In arsenic-induced neurodegenerative toxicity, arsenic promotes tau phosphorylation in the rat brain, possibly through activation of tau kinases, ERK12, JNK, and CDK5, which are associated with neurodegeneration [21]. ATO-induced developmental neurotoxicity (DNT) and induced antioxidant gene expression were negatively correlated with Glutathione levels [22]. ATO is a chemotherapy drug whose mechanism of ATO-induced hepatotoxicity has not yet been elucidated. Research has shown that ATO easily accumulates in liver cells, leading to cell membrane damage and liver enzyme leakage [23, 24]. The main component of hepatoprotective agents, sulfhydryl, easily combines with ATO, especially trivalent arsenicals [24]. Higher concentrations of sulfhydryl may weaken the role of iAsIII in promoting differentiation and reduce/delay hyperleukocytosis. Some interrelationships among the risk factors have been reported in the literature [25]. The risk factors in this study may be interrelated. For example, most patients receiving anti-leukocytosis agents have high WBC counts, so WBC count is a risk factor in the univariate analysis, but it is not an independent risk factor in the multivariate analysis. Therefore, ATO, prophylactic hepatoprotective agents and agents against leukocytosis may interact with each other in our study, which explains why some factors were found to be risk factors in the univariate analysis but not in the multivariate analysis.

The administration of anti-leukocytosis agents or prophylactic hepatoprotective agents is an independent risk factor for both overall hepatotoxicity and early hepatotoxicity. The incidence of ATO-induced hepatotoxicity (44/46, 95.65%) in patients who received agents against leukocytosis was higher than that of ATO (68/76, 89.47%), possibly because patients with high WBC counts account for majority of patients receiving anti-leukocytosis agents. Patients with high WBC counts are relatively high-risk patients, so they are more prone to ATO-induced hepatotoxicity; other drugs, such as anthracyclines, also have certain liver toxicity, and their combination with ATO can lead to liver toxicity. Therefore, the lack of other chemotherapeutic agents (single-agent arsenic trioxide treatment) might confer benefits. Whether the prophylactic application of hepatoprotective agents can independently predict the incidence of overall and early ATO-induced hepatotoxicity remains unclear. The enrolled patients did not receive any complementary medicine products and/or drugs that might induce hepatotoxicity during the ATO treatment process. In terms of elevated liver enzymes, the prophylactic application of hepatoprotective agents significantly reduces the incidence of ATO-induced hepatotoxicity. However, this prophylactic application must occur prior to the occurrence of hepatotoxicity, and there are currently no guidelines for the prophylactic application of hepatoprotective agents. The role of prophylactic application of hepatoprotective agents remains unknown.

The fibrinogen OR value was 3.496 (95% CI, 1.127–10.846). As an indicator of early ATO-induced hepatotoxicity, FIB ≤ 1 g/L was identified as an independent risk factor for early hepatotoxicity. The lower the FIB is, the higher the bleeding risk. To some extent, this association reflects abnormalities in coagulation, which may be related to liver coagulation factors and other related factors, thereby causing indirect hepatotoxicity. This needs to be further proven. Fibrinogen level is an independent risk factor for early ATO-induced hepatotoxicity, and D-D is an indicator of blood coagulation, but due to the inconsistencies in testing standards, effective statistical analyses cannot be performed. Although it is unclear whether ATO-induced hepatotoxicity is caused by liver cell injury, our study results may be useful for designing more appropriate risk stratification treatment protocols aimed at reducing ATO-induced hepatotoxicity.

WBC count, as a risk factor for early ATO-induced hepatotoxicity, was a statistically significant risk factor for the occurrence of early ATO-induced hepatotoxicity in this study. An increase in white blood cells affects the occurrence of liver injury, and the mechanism may be related to cytokines [26]. The induced differentiation of APL cells results in the secretion of IL-1β, IL-6, IL-8, TNFα, and other cytokines, and the increase in IL-1 has a parallel relationship with the number of peripheral blood leukocytes [27, 28]. In future studies, we will examine this topic. However, in the multivariate analysis, due to the nonindependent risk factors, most patients with high leukocytes have used agents to combat leukocytosis, and these agents are associated with the occurrence of ATO-induced hepatotoxicity.

In terms of predictive factors, a high hemoglobin level (HB ≥ 80 g/L) is a powerful indicator and could independently predict the occurrence of overall ATO-induced hepatotoxicity. In terms of the mechanism, it may be that arsenic metabolite concentrations are higher in red blood cells (RBCs) than in plasma [29], and the initial component of ATO and its metabolites are mainly metabolized by the liver, so the high HB level is an indirect response to the intake of ATO. HB was a powerful indicator for overall hepatotoxicity in this study, and the OR value (OR = 8.653; 95% CI, 1.339–55.921) was significantly higher than that for early hepatotoxicity (OR = 1.756; 95% CI, 0.662–4.659). However, there were no statistically significant differences in other factors, such as age, sex, and platelet count. The factors with less statistical significance may be indicators of the occurrence of ATO-induced hepatotoxicity in a larger sample size, which is a direction for future studies. Age, sex, and platelet count are confounding factors that are not independent risk factors for ATO-induced hepatotoxicity. Factors such as alcohol consumption, hemorrhagic disease, and chronic inflammatory disease may also contribute to confounding bias. In our study, we excluded or adjusted the analysis process.

The liver plays an important role in metabolizing drugs, especially chemotherapy agents, and hepatotoxicity often limits the delivery of the intended dose. ATO-induced hepatotoxicity involves ATO transformation and is also related to individual tolerance. Thus, improved identification of the predictive factors of hepatotoxicity is helpful. It is important to develop more reasonable therapeutic strategies and to maximize the role of ATO in the treatment of APL while reducing hepatotoxicity. To the best of our knowledge, this study is the largest sample size used in an analysis of ATO-induced hepatotoxicity. However, we also acknowledge several limitations inherent to this study. Further investigation is needed, including multicenter validation of ATO-induced hepatotoxicity, along with the development of new protective strategies to prevent hepatotoxicity.

## Data Availability

The data that support the findings of this study are available on request from the corresponding author, upon reasonable request.
